# Pulmonary lesions reshape swine respiratory microbiota: evidence of bacterial dysbiosis and reduced diversity

**DOI:** 10.1007/s42770-026-01981-1

**Published:** 2026-06-16

**Authors:** Kendra Rodeghiero, Gabriela Merker Breyer, Karine Ludwig Takeuti, Franciele Maboni Siqueira

**Affiliations:** 1 Laboratory of Veterinary Bacteriology, Federal University of Rio Grande do Sul, Porto Alegre, Brazil; 2 Feevale University, Campo Bom, Brazil

**Keywords:** Swine respiratory microbiota, Longitudinal microbiota, Lower respiratory tract (LRT), *16S-rRNA* gene sequencing, Candidate discriminant taxa

## Abstract

The swine lower respiratory tract (LRT) microbiota has increasingly been investigated, but important knowledge gaps remain, limiting our understanding of microbial communities involved in respiratory health and disease. This longitudinal study evaluated the LRT bacterial composition of pigs with pulmonary lesions (PL+) compatible with *Mycoplasma hyopneumoniae* infection and without pulmonary lesions (PL-) across production phases from nursery to slaughter. Using *16 S-rRNA* gene sequencing of intratracheal mucus and lung fragments, we observed longitudinal shifts in bacterial community composition. PL- animals showed higher observed richness and Shannon diversity during the finishing phases, whereas PL+ animals showed reduced alpha-diversity values and less pronounced temporal variation in relative composition. A total of 18 ASVs were detected across all sampled phases in both groups, suggesting a small set of persistent taxa; however, their prevalence, abundance, and ecological relevance remain unclear in the present analysis. Furthermore, LEfSe identified ASVs classified as *Tetragenococcus halophilus* and *Bdellovibrio bacteriovorus* as candidate discriminant taxa associated with PL + and PL- groups, respectively; these associations should be treated as preliminary and dataset-specific. Together, these findings suggest that pulmonary lesion status is associated with altered respiratory bacterial community patterns and support longitudinal approaches for investigating swine respiratory health.

**Supplementary Information:**

The online version contains supplementary material available at 10.1007/s42770-026-01981-1.

## Introduction

The porcine respiratory microbiota has been increasingly investigated, but important knowledge gaps remain, particularly regarding the lower respiratory tract (LRT) and the methodological standardization across studies. These gaps limit the interpretation of bacterial community patterns associated with respiratory health and disease [1–3]. The LRT of pigs harbors a distinct microbial community that is generally less diverse than that of the upper respiratory tract and is typically dominated by members of the phyla *Firmicutes*,* Proteobacteria*,* Bacteroidota*, and *Actinobacteriota*. Studies have shown that this community is more complex than initially thought, including genera such as *Streptococcus*, *Mycoplasma*, *Pasteurella*, *Veillonella*, *Lactobacillus*, *Clostridium* and *Prevotella* [4]. Representative species reported in the porcine respiratory tract include *Streptococcus suis*, *Streptococcus porci*, *Mycoplasma hyorhinis*, *Mycoplasma flocculare*, *Pasteurella multocida*, *Veillonella caviae*, *Lactobacillus amylovorus*, *Glaesserella indolica*, and *Clostridium perfringens* [4, 5]. Increasing evidence indicates that alterations in this microbial community are associated with respiratory diseases and pneumonia in pigs, suggesting that disruptions in the respiratory microbiota may influence pathogen colonization and disease progression [4, 5].

Respiratory microbial diversity has been associated with host immune responses and susceptibility to respiratory infections, although causal relationships remain incompletely defined [2–4]. Previous studies have reported changes in the LRT microbiota composition in piglets with lung lesions compared to healthy animals [5, 6]. These modifications may negatively affect animal health, contributing to the development of respiratory diseases and generating economic impacts on animal production [1]. Swine enzootic pneumonia, primarily caused by *Mycoplasma hyopneumoniae*, is one of the most important respiratory diseases in pigs worldwide. Its clinical severity is often influenced by coinfections with other bacterial agents commonly involved in the porcine respiratory disease complex (PRDC), such as *Pasteurella multocida*, *Mycoplasma hyorhinis*, *Streptococcus suis*, *Glaesserella parasuis*, *Actinobacillus pleuropneumoniae*, *Bordetella bronchiseptica*, and *Trueperella pyogenes*. Viral agents such as swine influenza virus, porcine reproductive and respiratory syndrome (PRRSV), and porcine circuvirus 2 (PCV2) may also participate in PRDC depending on regional epidemiology and herd status. Macroscopic lesions compatible with *M. hyopneumoniae*-associated enzootic pneumonia are often observed at slaughter and are considered indicators of chronic respiratory problems [7, 8].

Longitudinal studies are particularly valuable in microbiome research because they capture temporal dynamics of microbial communities, allowing the identification of microbial trajectories and host–microbiome interactions associated with health, disease, and life stages [9, 10]. Therefore, this study aims to evaluate longitudinal changes in the respiratory microbiota of pigs from nursery to slaughter and to compare the bacterial composition of animals with pulmonaxry lesions (PL+) compatible with enzootic pneumonia*/M. hyopneumoniae-*associated lesions infection with those without lesions (PL-), in order to identify potential associations between lung pathology and bacterial community structure.

## Materials and methods

### Experimental design and sampling

A nursery pig farm with 2,000 animals and a finishing herd with 600 pigs were selected for the longitudinal analysis. Both farms were commercial herds operating under routine veterinary supervision. Monitoring of *Actinobacillus pleuropneumoniae* was performed semiannually through the collection of approximately 60 serum samples for the detection of antibodies against the agent using a commercial ELISA kit. Since the serological results were negative and there were no clinical signs or lesions compatible with *A. pleuropneumoniae* infection, the herd was classified as negative. The PRRS status was interpreted in accordance with the official Brazilian sanitary classification, and no evidence of infection was detected in the reviewed herd records [11]. In the farms studied, *M. hyopneumoniae* infection and Influenza A virus circulation were considered established based on herd history, clinical signs, histopathological and molecular testing, and slaughterhouse findings.

The immunization protocol of the farrow-to-wean farm was based on vaccination of sows against enteric pathogens (*Escherichia coli*, *Clostridium perfringens*, and *Clostridioides difficile*), reproductive agents (Parvovirus and *Leptospira* sp.), and *M. hyopneumoniae*, PCV2, *Glaesserella parasuis*, *Pasteurella multocida*, and *Bordetella bronchiseptica*. Piglets at 21 days of age were immunized with commercial vaccines against *M. hyopneumoniae* and PCV2, as well as an autogenous vaccine against *G. parasuis* and *P. multocida*.

**Fig. 1:**
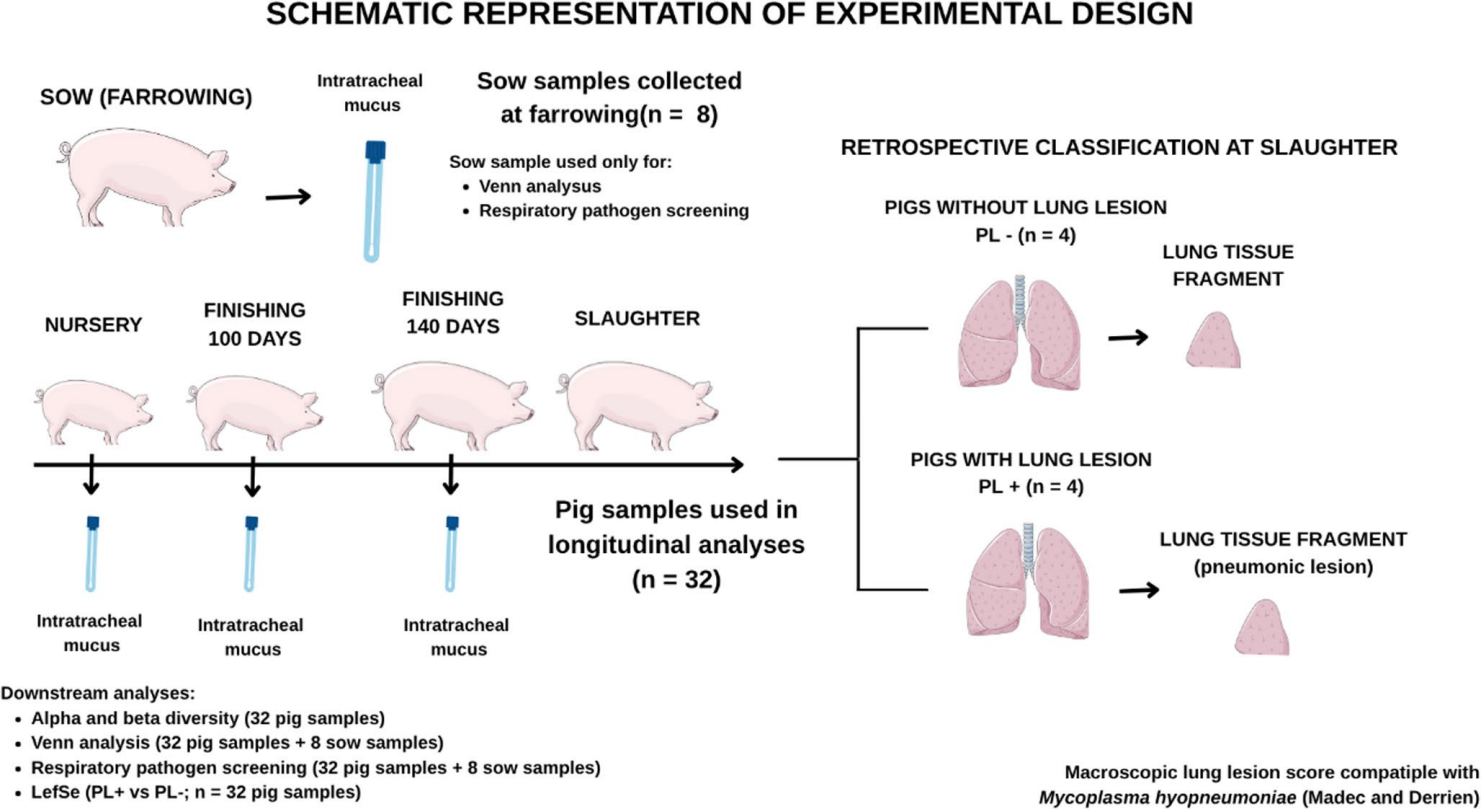
Schematic representation of experimental design. Eight animals were selected at slaughter and classified based on macroscopic lung lesion scoring compatible with *Mycoplasma hyopneumoniae* [8], as pigs with lesions (PL+, *n* = 4) or without lesions (PL−, *n* = 4). From these animals, samples collected at different production stages were evaluated, including nursery (intratracheal mucus), finishing at 100 and 140 days (intratracheal mucus), and slaughter (lung fragment). Additionally, intratracheal mucus samples were collected from the sows that farrowed each piglet

Eight piglets were randomly selected in the nursery, ear-tagged, and followed longitudinally until slaughter. Samples were collected at four stages: nursery (63 days), finishing (100 days), finishing (140 days), and slaughter (180 days). Sampling sites varied according to age and included intratracheal mucus during nursery and finishing phases and lung fragments at slaughter, as described by Takeuti et al. [12]. Intratracheal mucus samples were also collected from the sows that farrowed each piglet (Fig. 1).

It is worth mentioning that samples from sows were only included in the Venn analysis and respiratory pathogen screening (*n* = 40). Comparative analyses between PL- and PL+ only included samples from nursery and finishing piglets (*n* = 32).

To evaluate the influence of lung lesions on the respiratory microbiota, lungs were macroscopically examined at slaughter for lesions compatible with *M. hyopneumoniae*, according to Madec and Derrien [8]. Animals with score lesion 0 were classified as without lesion, and animals with lesion scores ranging from 1 to 4 were classified as having lung lesions. Eight animals were selected and classified as pigs with lesions (PL+; *n* = 4) or without lesions (PL−; *n* = 4). Samples collected in the previous phases were then retrospectively grouped according to this classification, resulting in a total of 32 samples analyzed.

### Partial 16 S-rDNA gene sequencing

The samples underwent total bacterial DNA extraction using the PureLink^®^ Genomic DNA Mini Kit (Invitrogen) according to the manufacturer’s recommendations. The extracted DNA was stored at -80 °C for subsequent analysis.

To determine the bacterial community in the samples, the V4 region of the 16 S-rDNA gene was targeted for next-generation sequencing using a protocol optimized for samples with low bacterial load [13]. Specifically, a nested PCR was performed for each sample using the universal primers 515 F and 806R [14], containing Illumina adapters. In the first reaction, 12.5 ng of metagenomic DNA was used as the template; and in the second reaction, 5 µL of the previous amplicon was used. In parallel, blank community were processed alongside all samples, and PCR negative controls were included. The integrity of the amplified DNA was verified by electrophoresis, confirming the presence of bands on a 1% agarose gel.

Subsequently, the obtained amplicons underwent paired-end 2 × 250 bp sequencing on the Illumina MiSeq platform (Illumina, California, USA), following the manufacturer’s instructions (*MiSeq Reagent Kit v2*, 500 cycles).

### Metataxonomic and statistical analyses

The quality of raw sequences was assessed using FastQC (v0.11.9). Low-quality reads (Phred < 30), short sequences (< 50 nt), and primer and adapter sequences were removed using Trimmomatic [15]. Paired-end reads were processed in QIIME2 v2020.2 [16] using the with the q2-dada2 plugin [17] for read merging, quality filtering, chimera removal, and inference of amplicon sequence variants (ASVs), using truncation lengths of 150 bp for forward reads and 130 bp for reverse reads. Taxonomic assignment was performed using the Silva 138.1 database (classifier silva-138-99-515-806- nb-classifier.qza, 2022 version) [18].

Bacterial community analyses were conducted in RStudio v4.2.2 using the phyloseq package [19, 20]. Sequences classified as Eukaryota, Archaea, or unknown were removed prior to downstream analyses. Alpha diversity was estimated observed richness and Shannon diversity indices and compared using Kruskal–Wallis tests with pairwise Wilcoxon comparisons tests with p-values adjusted using the Benjamini–Hochberg method (*p* < 0.05). Beta diversity was evaluated using Bray–Curtis distances, visualized by principal coordinate analysis (PCoA) [21], and statistically tested using PERMANOVA with 999 permutations (*p* < 0.05) in the vegan package [20]. Homogeneity of multivariate dispersion was assessed using betadisper followed by permutation testing (permutest).

Comparisons were performed between animals with (PL+) and without (PL–) pulmonary lesions and across production phases (nursery, finishing at 100 days, finishing at 140 days, and slaughter), excluding the sow group. Community composition was visualized using barplots of the 30 most abundant genera and Venn diagrams illustrating shared ASVs between phases and lesion groups. Venn diagrams were used only to summarize shared ASV detection across groups/phases and were not interpreted as abundance or prevalence measures.

A Sankey plot was generated to visualize the distribution flow of bacterial phyla across the production phases (nursery, finishing at 100 days, finishing at 140 days, and slaughter). Relative abundances were summarized at the phylum level using phyloseq and dplyr [19, 21], including the five most abundant phyla, and visualized using ggsankey [22] and ggplot2 [23].

To further investigate the presence of relevant bacterial taxa, the total ASV abundance of genera containing species commonly associated with respiratory pathogens involved in swine respiratory infections was examined, including the genera *Mycoplasma*, *Actinobacillus*, *Bordetella*, *Erysipelothrix*,* Pasteurella*, *Streptococcus*, and *Glaesserella* [24, 25]. Finally, candidate discriminant taxa associated with lesion status were identified using the LEfSe method (Linear Discriminant Analysis Effect Size) [26], with a significance threshold of *p* < 0.05 and an LDA score > 2.0. Data normalization was performed using the microbiome Marker package [27].

## Results

Sequencing summary. Samples were sequenced targeting the V4 region of the 16 S rRNA gene. After quality filtering and demultiplexing, a total of 928,378 sequences were retained for downstream analyses (mean = 29,012 ± 43,979 reads per sample). The median read length was 254 bp. Overall, 1,907 ASVs were identified and used in subsequent analyses. Raw sequencing read counts for each sample are provided in Supplementary Table S1 as sequencing-depth information; these values should not be interpreted as absolute bacterial load.

### The bacterial population in the lower respiratory tract changes over the course of pigs’ lives

**Fig. 2:**
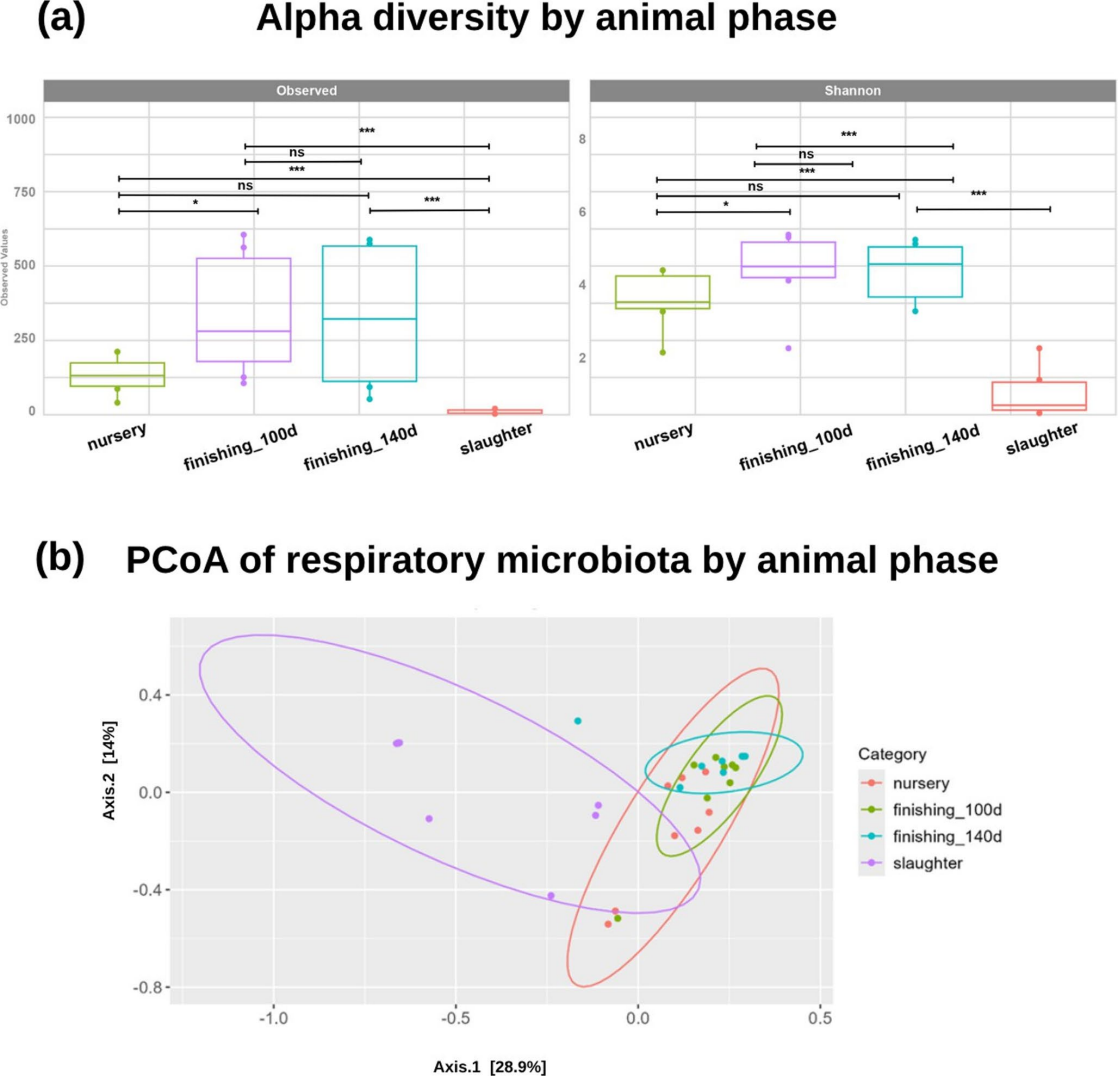
Respiratory bacterial diversity in pigs at different developmental stages (nursery, finishing at 100 and 140 days, and slaughter). (**a**) Alpha diversity using Observed richness and Shannon diversity indices with statistical analysis performed using the Kruskal–Wallis test (*p* < 0.05). (**b**) Principal coordinates analysis (PCoA) based on Bray-Curtis distances showing the distribution of samples from the nursery (red), finishing at 100 days (green), finishing at 140 days (blue), and slaughter (purple)

Alpha diversity analyses (Fig. 2A) showed higher observed richness and Shannon diversity during the finishing phases (100 and 140 days) than during nursery and slaughter (*p* < 0.05). Samples collected at slaughter displayed the lowest alpha diversity values among the stages (Fig. 2A). Bray-Curtis PERMANOVA did not indicate significant differences in community structure among groups when sow samples were excluded (F = 1.2109, R² = 0.0388, *p* = 0.206). Homogeneity of multivariate dispersion did not differ among groups (betadisper/permutest, *p* = 0.239), supporting the absence of group differences. In the PCoA, slaughter samples tended to separate from the other phases, whereas nursery and finishing samples showed substantial visual overlap in the ordination.

To determine the shifts in bacterial composition of the pigs’ LRT over their lives, a descriptive longitudinal analysis was performed to visualize within-animal changes across stages, from the nursery to slaughter, in a longitudinal analysis.

**Fig. 3:**
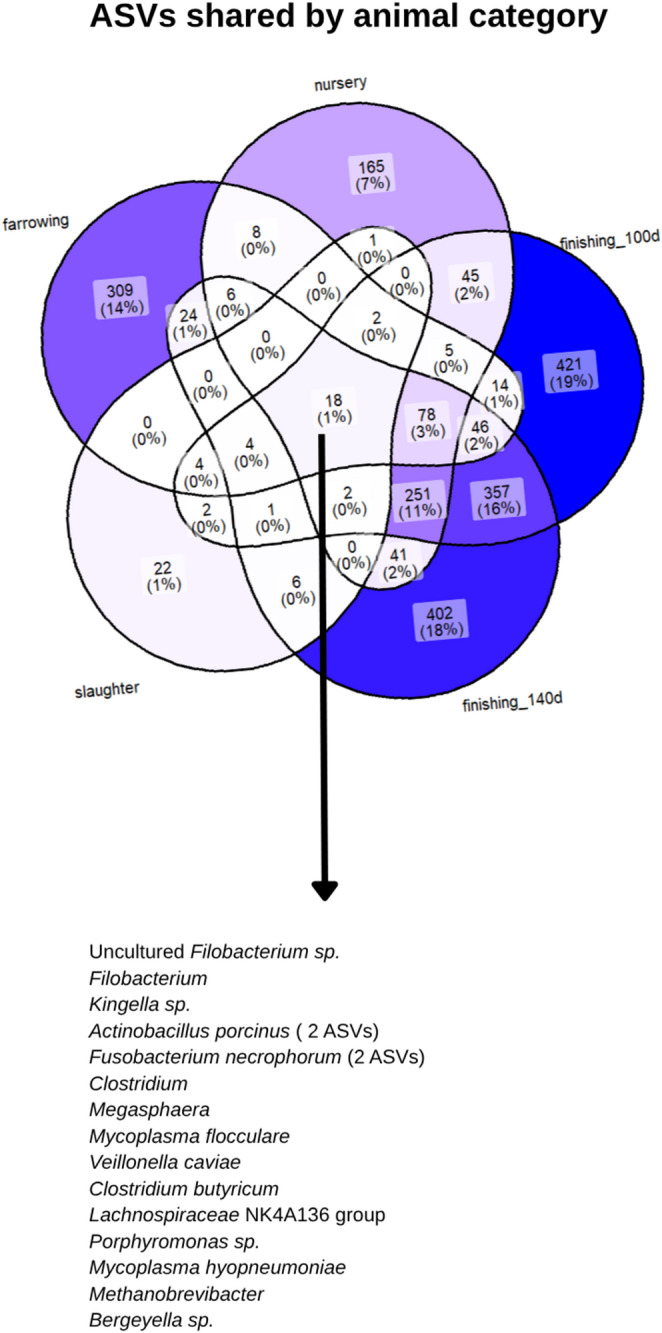
Shared ASVs across sampled groups, including sow samples and pig production phases. Venn diagram of ASVs in farrowing, nursery, finishing 100 days, finishing 140 days, and slaughter. ASVs shared across all stages represent 1% of the total ASVs. The corresponding taxa of these shared ASVs are listed below the diagram. Shared ASVs indicate persistence across production stages but do not reflect their relative abundance or prevalence within each phase

To understand how ASVs persist throughout the animal’s life in a longitudinal context, a Venn diagram was generated to identify ASVs that are unique and shared across the swine production stages. Distinct patterns of ASV sharing and uniqueness were detected among the five analyzed stages (Fig. 3). The farrowing phase (corresponding to the sows) had 309 (14%) unique ASVs, the nursery phase had 185 (7%) unique ASVs, the finishing phase at 100 days of age had the highest number of unique ASVs with 421 (19%), followed by the finishing phase at 140 days with 402 (18%). The slaughter phase showed the lowest number of unique ASVs, with only 22 (1%).

Regarding the ASVs shared across all phases (18 ASVs – 1%) (Fig. 3), these taxa represent a small subset of the microbiota that persists throughout the production cycle. However, it is important to note that this analysis reflects only the presence of these ASVs across stages and does not provide information on their relative abundance or prevalence within each phase. Among the shared ASVs, some potentially pathogenic bacteria were identified, such as ASVs classified as *Actinobacillus porcinus*, *Fusobacterium necrophorum*, *M. hyopneumoniae*, as well as taxa commonly associated with the respiratory microbiota were also found, including ASVs classified as *Veillonella caviae*, *Clostridium butyricum*, *Megasphaera elsdenii*, and *Mycoplasma flocculare*. These findings suggest that a limited set of ASVs was persistently detected across production phases, although their ecological relevance, abundance, and prevalence remain unclear from the present analysis.

**Fig. 4:**
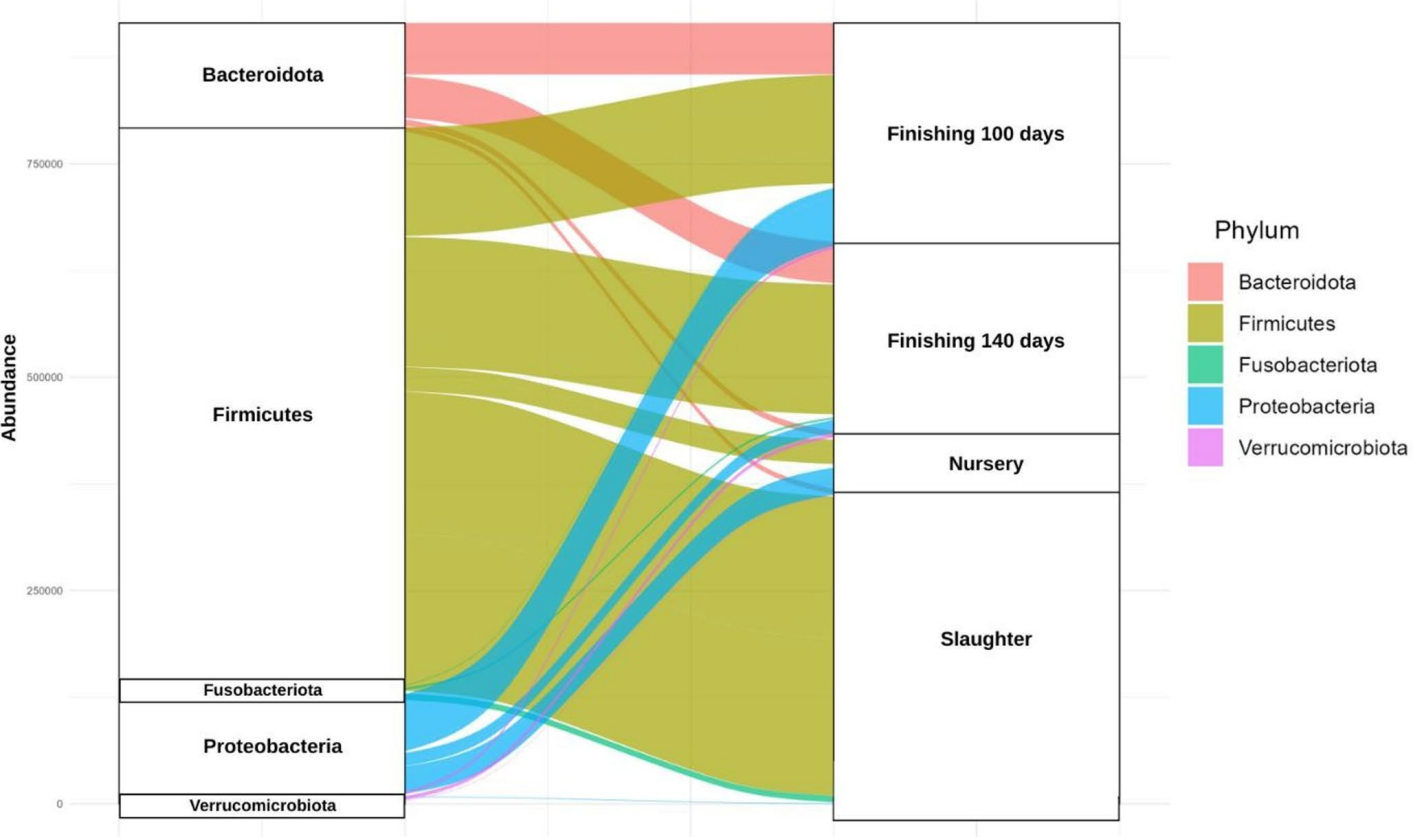
Distribution and flow of the major bacterial phyla across the production phases (nursery, finishing at 100 days, finishing at 140 days, and slaughter). The width of each flow represents the relative abundance of each phylum within and across phases

The Sankey plot illustrates the distribution and dynamics of the major bacterial phyla across production phases (Fig. 4). *Firmicutes* was the dominant phylum across all stages, contributing most extensively throughout the production cycle, particularly during the finishing phases (100 and 140 days). *Proteobacteria* was the second most abundant phylum, showing relatively higher abundances in nursery and slaughter than in finishing stages. *Bacteroidota* contributed at lower levels and was primarily associated with the finishing stages. *Fusobacteriota* and *Verrucomicrobiota* remained minor components.

#### Association between pulmonary lesions and the lower respiratory microbiota of swine with lung lesions

**Fig. 5:**
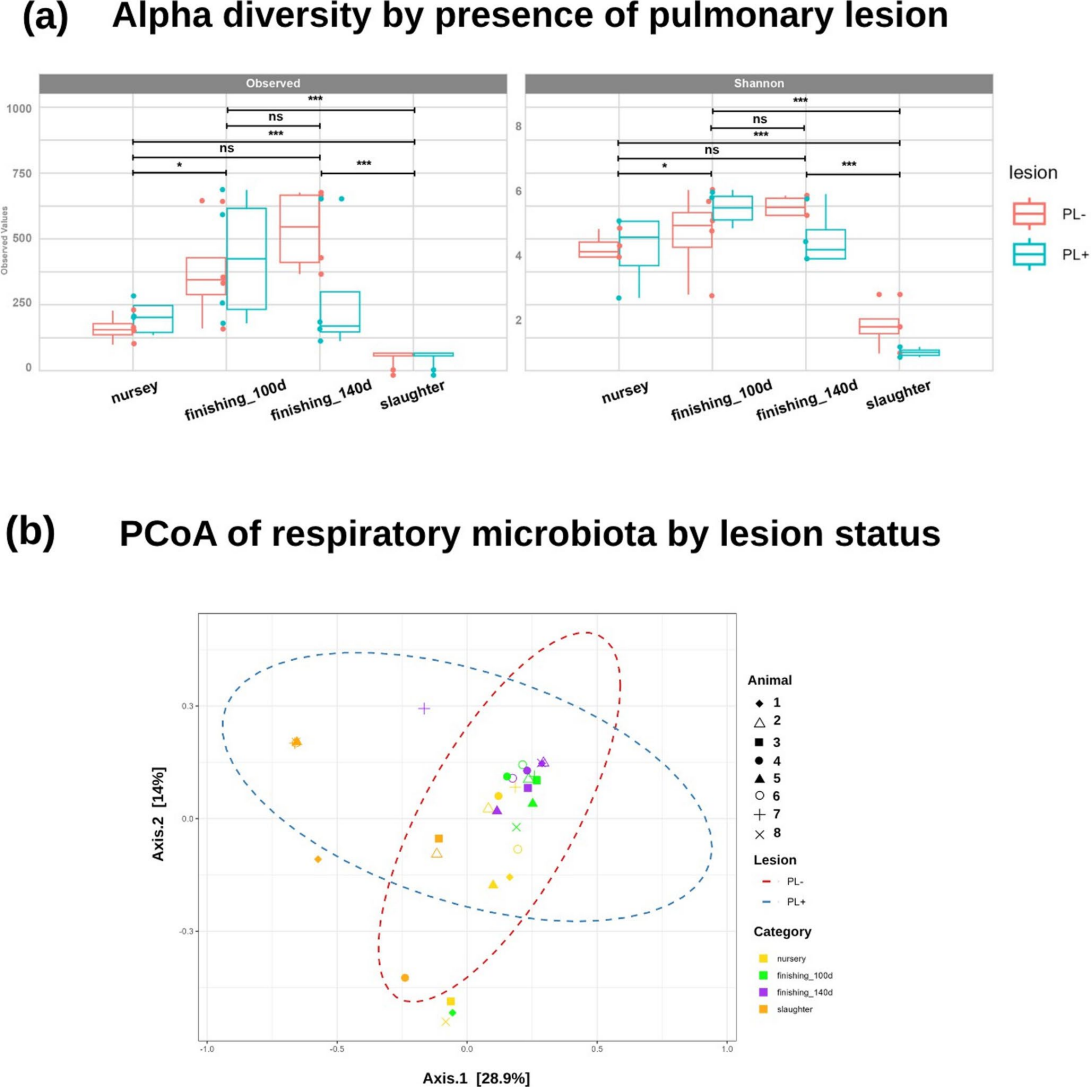
Comparison of respiratory microbiota between pigs with (PL+) and without (PL−) lung lesions across production stages. (**A**) Alpha diversity, represented by observed richness and Shannon diversity indices. Statistical differences between groups were assessed using the Wilcoxon test (*p* < 0.05). (**B**) Principal coordinates analysis (PCoA) based on Bray–Curtis distances showing the distribution of PL + and PL− samples, indicating partial overlap between groups. Group differences were assessed by PERMANOVA and dispersion by betadisper/permutest

Figure 5A suggests that PL- animals had higher observed richness and Shannon diversity than PL+ animals during the finishing phases (100 and 140 days), whereas both groups showed markedly reduced alpha-diversity values at slaughter. In contrast, Fig. 5B indicates partial overlap between PL + and PL− samples in the Bray–Curtis ordination. Bray–Curtis PERMANOVA did not indicate significant differences in community structure between groups (F = 1.052, R² = 0.0339, *p* = 0.338). Homogeneity of multivariate dispersion did not differ between groups (betadisper/permutest, *p* = 0.239). These results do not support significant differences in community structure associated with lesion status, and the observed ordination patterns should therefore be interpreted cautiously.

**Fig. 6:**
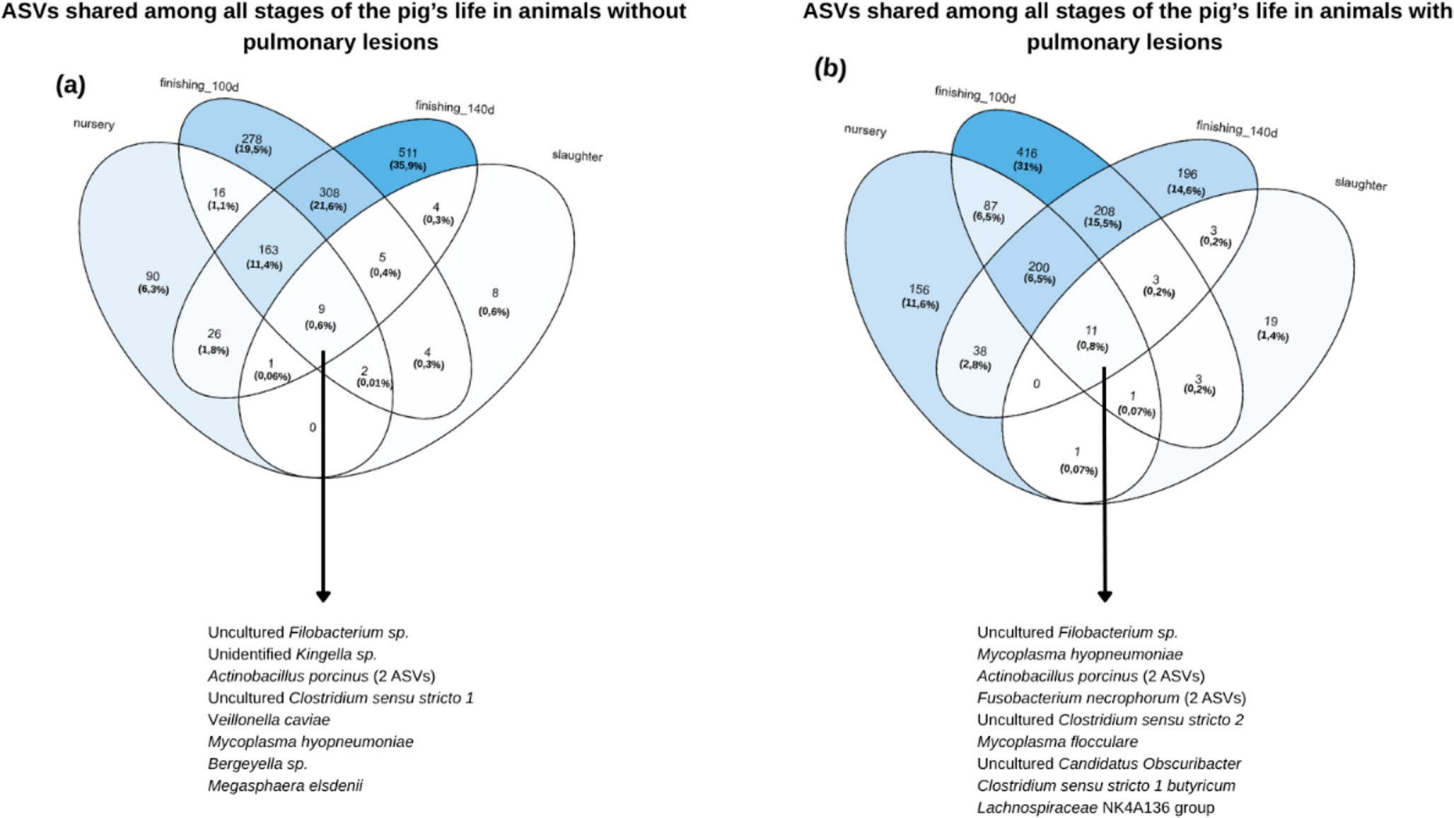
ASVs distribution across pigs’ lifespan from nursery to slaughter in animals with (PL+) and without lung lesions (PL-). (**A**) Venn diagram of ASVs identified in the different swine production phases in the group without lung lesions. Box showing the ASVs shared across all stages, corresponding to 0.6% of the total ASVs. (**B**) Venn diagram of distribution of ASVs identified in the different swine production phases in the group with lung lesions. The corresponding taxa of these shared ASVs are listed below each diagram. Shared ASVs indicate persistence across production stages but do not reflect their relative abundance or prevalence within each phase. The taxa listed below each diagram correspond to ASVs in the central intersection shared by all sampled stages within that group

The Venn diagram demonstrated patterns of ASV sharing and uniqueness between the animals with and without lung lesions (Fig. 6). In PL- group (Fig. 6A), animals at finishing (140 days) showed the highest number of unique ASVs (35.9%), while the slaughter phase showed the lowest number (0.6%). Less than 1% of the ASVs were shared across all animals without lesions, corresponding to the central intersection of the Venn diagram. These shared ASVs represent a small subset of taxa that persist throughout the production cycle; however, it is important to note that this analysis reflects only their presence across stages and does not provide information on their relative abundance or prevalence. Among the shared ASVs, which persist across all tested production phases, we highlight ASVs classified as *M. hyopneumoniae* and *A. porcinus*, both bacteria with pathogenic potential.

In the PL+ group (Fig. 6B), the phase with the highest number of unique ASVs was the 100-day finishing phase, accounting for 31% of the total ASVs. Conversely, the slaughter phase showed the lowest number of unique ASVs, representing 1.4% of the total. ASVs shared across all phases, represented in the central intersection of the Venn diagram, included taxa classified as *M. hyopneumoniae*, *A. porcinus*, and *F. necrophorum*. Their detection indicates persistence across sampled stages but does not establish their abundance, prevalence, or role in lesion development (Fig. 6A and B).

**Fig. 7:**
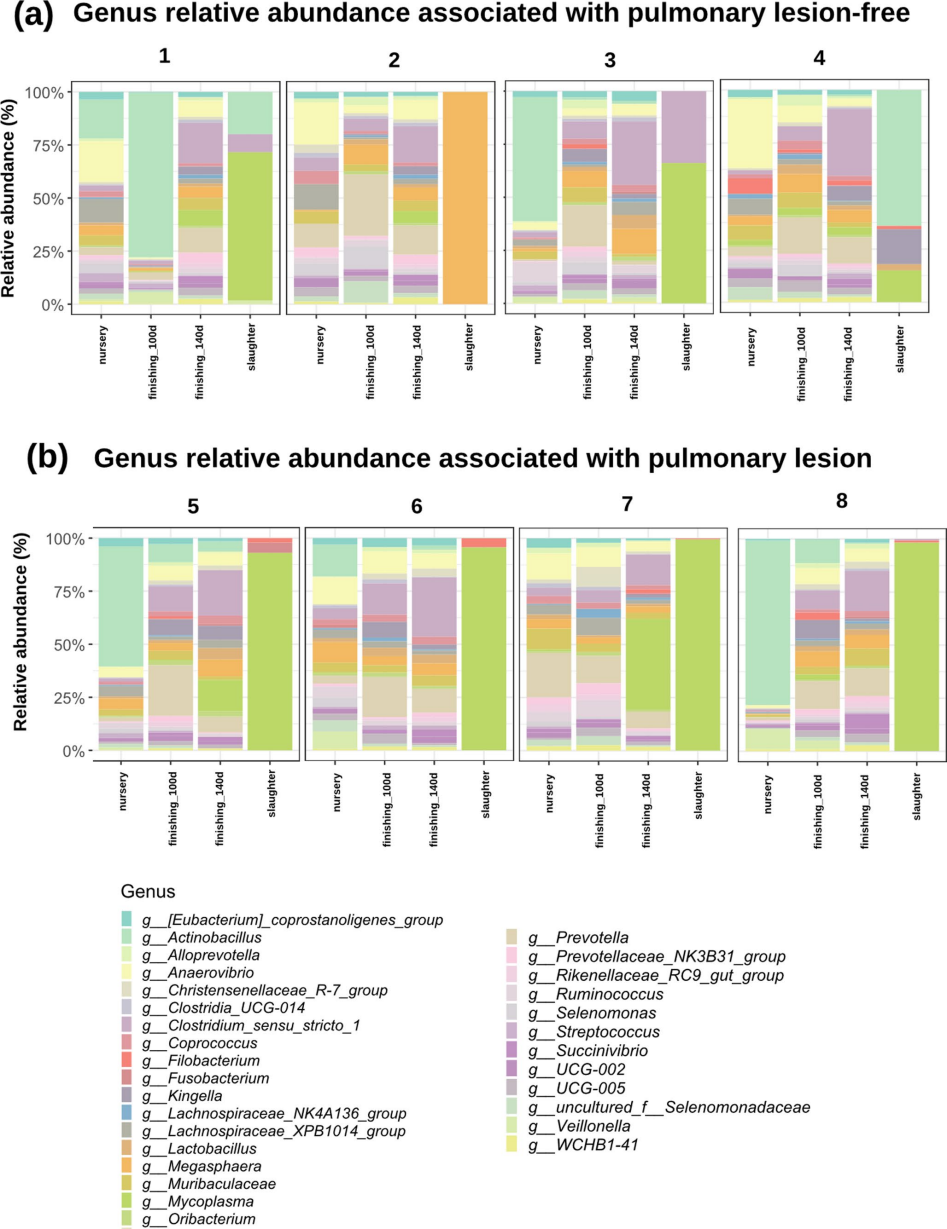
Bacterial relative abundance in the lower respiratory tract of swine. (**A**) Composition of the group without lung lesions; (**B**) Composition of the group with lung lesions. Relative abundance was used to describe community composition across samples with variable sequencing depth and bacterial load

Genus-level relative abundance profiles showed descriptive variation among individuals and production phases (Fig. 7). In PL- animals, the relative composition appeared more heterogeneous across individuals, with several genera contributing to the profiles, including *Prevotella*, *Lactobacillus*, *Ruminococcus*, members of *Christensenellaceae* family and *Anaerovibrio*. In PL+ animals, slaughter samples were characterized by a higher relative contribution of *Mycoplasma*-related reads. Across production stages, compositional patterns varied within each group, although these differences were descriptive rather than statistically tested. Because these data are based on relative abundance from amplicon sequencing, they should be interpreted as compositional patterns rather than absolute changes in bacterial load.

### Investigation of the presence of respiratory pathogens related to the Porcine Respiratory Disease Complex in the tested animals

To screen for genera containing species associated with PRDC [28], specific genera were investigated, including *Actinobacillus*, *Erysipelothrix*, *Mycoplasma*, *Pasteurella*, *Streptococcus*, *Bordetella*, and *Glaesserella*. Their presence was evaluated across different life stages and in the respective sows, as shown in Table 1.

Higher read counts assigned to *Actinobacillus* reads was observed in the nursery and finishing (100 and 140 days) phases, as well as in the dams of all animals. Furthermore, *Mycoplasma*-assigned reads were more prominent at slaughter, which agrees with the relative abundance results. In contrast, a reduced number of reads for this same pathogen was observed in the sows.

**Table 1:** Sequencing reads assigned to genera containing species associated with Porcine Respiratory Disease Complex (PRDC)

	Pathogens^1^						
		*Actinobacillus* (reads)		*Erysipelothrix* (reads)	*Mycoplasma* (reads)	*Pasteurella* (reads)	*Streptococcus* (reads)
Animal	Pulmonary lesion	Phase					
		Sow	63,481	0	3	48	128
		Nursery	1137	0	0	0	257
1	No	Finishing_100d	50,402	0	15	43	1311
		Finishing_140d	117	0	3529	63	88
		Slaughter	61	0	214	0	0
		Sow	36,358	0	0	0	0
		Nursery	0	0	0	0	6
2	No	Finishing_100d	30	0	17	11	70
		Finishing_140d	180	0	3454	36	123
		Slaughter	0	0	0	0	0
		Sow	107	0	21	0	67
		Nursery	1837	0	0	0	79
3	No	Finishing_100d	393	7	0	47	132
		Finishing_140d	380	0	89	0	416
		Slaughter	0	0	121	0	0
		Sow	5570	0	3	36	56
		Nursery	1837	0	0	0	79
4	No	Finishing_100d	393	7	0	47	132
		Finishing_140d	380	0	89	0	416
		Slaughter	0	0	121	0	0
		Sow	401	0	25	0	66
		Nursery	3666	0	0	0	155
5	Yes	Finishing_100d	3482	19	235	0	378
		Finishing_140d	44	0	136	0	0
		Slaughter	22	0	144,492	0	0
		Sow	25,304	4	7	0	0
		Nursery	1231	0	42	26	46
6	Yes	Finishing_100d	0	0	0	53	50
		Finishing_140d	50	0	0	0	47
		Slaughter	18	0	15,254	0	0
		Sow	400	0	0	32	135
		Nursery	0	0	18	0	21
7	Yes	Finishing_100d	0	0	23	16	34
		Finishing_140d	0	0	1258	0	5
		Slaughter	0	0	6554	0	0
		Sow	17,403	0	0	0	112
		Nursery	22,975	0	0	0	212
8	Yes	Finishing_100d	599	0	142	17	31
		Finishing_140d	171	0	86	35	198
		Slaughter	0	0	182,239	0	203

^1^Genus-level assignment does not confirm pathogenic species identity

### Candidate discriminant taxa associated with lesion status

**Fig. 8:**
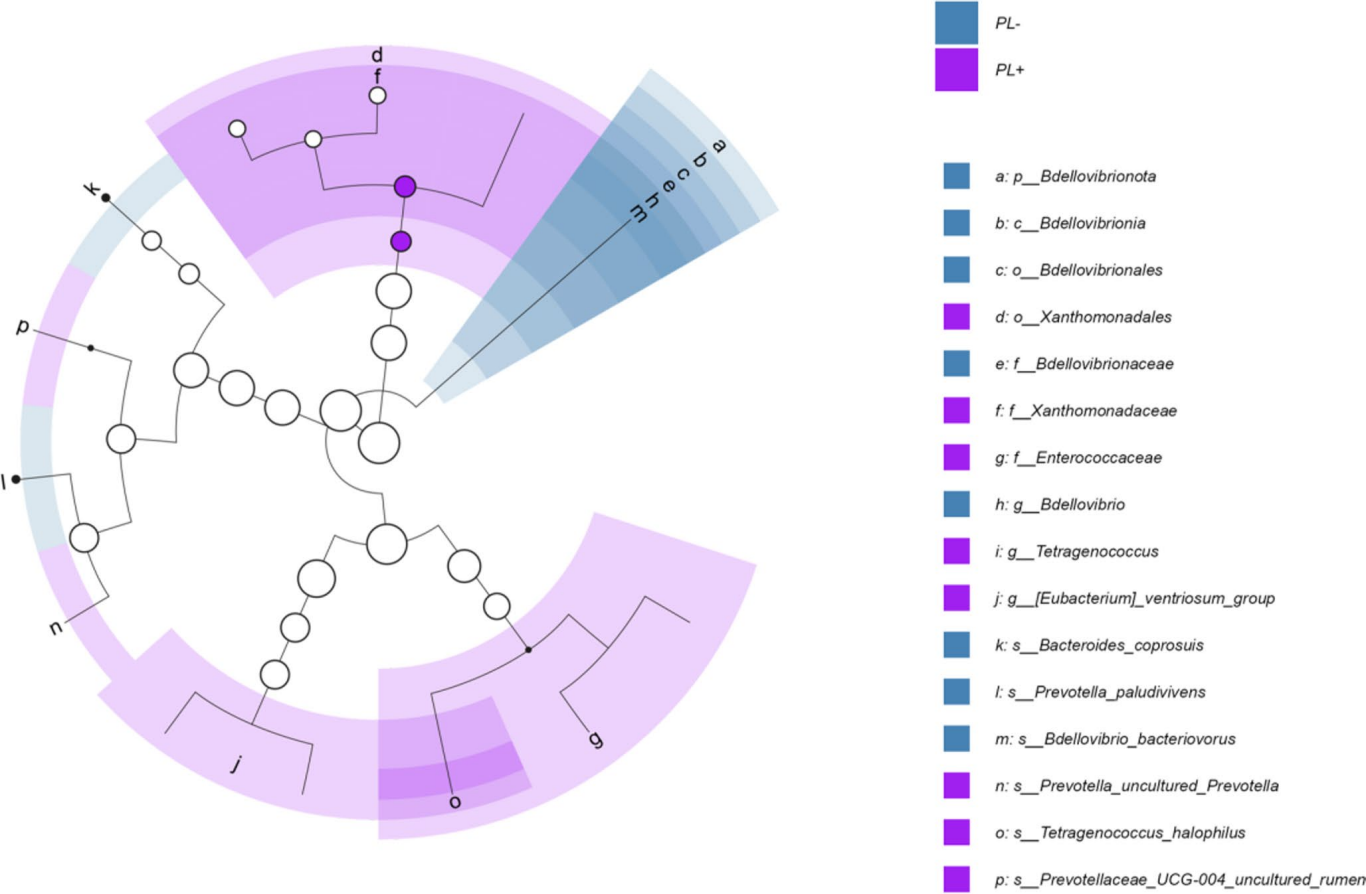
Differentially abundant taxa between pigs with (PL+) and without (PL-) lung lesions identified using LEfSe (Linear Discriminant Analysis Effect Size). Data were normalized using the microbiomeMarker package prior to analysis. Statistical significance was set at *p* < 0.05, and candidate discriminant taxa associated with lesion status were retained at a logarithmic LDA score threshold > 2.0. The cladogram represents the taxonomic hierarchy of discriminant taxa, with clades highlighted in purple indicating taxa enriched in PL+ animals and clades highlighted in blue indicating taxa enriched in PL− animals. Filled circles indicate taxa with statistically significant differences, and color intensity reflects the magnitude of the association between each taxon and the respective group

The search for biomarkers related to the presence and absence of lung lesions among individuals with and without lung lesions was investigated. Figure 8 presents the differentially abundant taxa between the groups with lesions (PL+) and without lung lesions (PL-), indicating candidate discriminant taxa associated with lesion status.

In PL+ group, *Xanthomonadales* (*p* < 0.05), as well as the family *Enterococcaceae* and the species *Prevotella* spp. and ASVs classified as *Tetragenococcus halophilus* showed marked abundance (Fig. 8). *T. halophilus* stands out with a high magnitude of association with the presence of lung lesions in swine, according to the LEfSe analysis.

Conversely, in PL- group, the presence of ASVs classified as *Prevotella paludivivens (p < 0.05*) and *Bacteroides coprosuis (p < 0.05)* is notable (Fig. 8). *Bdellovibrio bacteriovorus* showed a significant association with the absence of lung lesions (Fig. 8). In this dataset, LEfSe identified ASVs classified as *T. halophilus* and *B. bacteriovorus* as candidate discriminant features associated with PL + and PL- groups, respectively.

## Discussion

Respiratory microbial community composition has been associated with host immune responses and respiratory disease outcomes in pigs, although causal mechanisms remain incompletely defined. However, the swine respiratory microbiota remains incompletely characterized, particularly in the LRT. Several studies have explored its bacterial composition [1, 2], but important gaps remain regarding the structure and function of these microbial communities that support respiratory health or contribute to disease [1–3]. In this context, the present study investigated the respiratory microbiota of swine longitudinally, comparing the bacterial composition of the LRT from the nursery to slaughter in animals with and without pneumonic lesions. This approach aimed to provide a broader view of the dynamic changes in the microbiota throughout the production cycle, allowing the identification of candidate taxa associated with lesion status and longitudinal community patterns. The results demonstrated changes in the microbiota composition throughout the animals’ life, as well as differences between animals with and without lung lesions. These patterns may reflect multiple factors, including animal management practices, antimicrobial use, stress, and other challenges during transitions between production phases.

One limitation when comparing the phases is the difference in sample types. In detail, at nursery and finishing (100 and 140 days), intratracheal mucus was collected; and at slaughter, lung fragments with pneumonic lesions were collected. These differences in sample matrix may influence the observed microbial composition, as distinct anatomical sites and tissue types can harbor different microbial communities. Therefore, differences observed at slaughter may reflect both biological stage and sample-matrix effects. However, when comparing samples of intratracheal mucus from the nursery, and the finishing phases at 100 and 140 days, clear changes in the LRT microbiota composition were observed.

Each phase also exhibited exclusive ASVs (Fig. 3), suggesting that different life stages favored the colonization by specific genus. Pigs in the finishing phase at 100 days had a higher number of unique ASVs (19%), this pattern may be related to stage-specific exposures or management factors, although the present study was not designed to test these drivers directly. Figure 3 revealed the presence of a small set of shared ASVs, representing 1% of all detected ASVs, across all production phases analyzed (including both pigs and their sow). This finding suggests that some taxa persisted across sampled phases, although their ecological role in the LRT remains unclear. ASVs classified as *Filobacterium*, *F. necrophorum*, and *A. porcinus* were each represented by two distinct ASVs and were therefore identified in duplicate. Overall, the flow patterns highlight a consistent predominance of *Firmicutes* across the production cycle, with variation in *Proteobacteria* relative abundance across phases. These descriptive patterns may indicate stage-associated changes but do not directly measure community stability.

When analyzing bacterial diversity (Fig. 5A and B), a reduction in alpha diversity was observed in pigs with lung lesions (PL+). The lower alpha diversity observed in PL+ animals suggests an association between lesion status and altered respiratory microbiota. However, the study design does not determine whether these patterns are a cause or consequence of pulmonary lesions. The reduced diversity in these animals could represent either a consequence of the lesion or a predisposing factor that favored the establishment of the bacterial community identified in this group. The partial overlap of the ellipses in PCoA indicates that not all PL+ animals share the same bacterial composition, and the intermediate region represents samples with a mixed community composition. In the study conducted by Wang et al. in 2018 [29], which compared the oropharyngeal microbiota composition of healthy piglets and piglets with respiratory disease, a potential association of the genera *Moraxella*, *Veillonella*, and *Porphyromonas* with respiratory diseases was indicated. In contrast, genera such as *Streptococcus*, *Prevotella*, *Neisseria*, and *Actinobacillus* were suggested to be part of the normal microbiota composition of these animals. Although *Actinobacillus* was detected across phases and groups, its role in the lower respiratory microbiota cannot be defined from these data alone; the same caution applies to *Filobacterium* (Fig. 5).

Among the ASVs shared across all production phases in pigs without lesions (Fig. 6A), the presence of ASVs classified as *Clostridium butyricum* stands out. This species is known for its probiotic effects in the intestinal tract, helping maintain gut microbiota homeostasis and contributing to immune modulation [30, 31]; however, its role in the respiratory microbiota remains unclear. We also highlight the presence of *Lachnospiraceae* across all phases in the PL+ group (Fig. 6B); Although some members of this family are linked to fermentation-related metabolic activities [32], their functional significance in the swine respiratory tract remains unclear. The ASVs classified as *A. porcinus* and *F. necrophorum* were each represented by two distinct ASVs and were therefore identified in duplicate.

When comparing the ASVs shared between both groups (PL + and PL-), ASVs classified as *M. hyopneumoniae* were present in both animals with and without lesions, as previously demonstrated by Siqueira et al. [5]., who compared the lung microbiome of animals with and without enzootic pneumonia. This supports that the presence of this bacterium alone is not sufficient to explain the occurrence of disease. Figure 7 shows a higher relative contribution of *Mycoplasma*-assigned reads in some samples; however, this result does not imply that a major PRDC associated agent is causing disease in the host. Among the pigs evaluated in our study, two *Mycoplasma* species were identified in the lungs: ASVs classified as *M. hyopneumoniae* (an agent associated with porcine enzootic pneumonia) and *M. flocculare*, which has not been reported as a disease-causing agent to date. The presence of *Mycoplasma* in the swine lung can alter the number of bacterial species present, as demonstrated in the study conducted by Almeida et al. [6].

Bacterial genera typically considered native to oral and gastrointestinal sites, such as *Prevotella* and *Lachnospiraceae*, were also identified in LRT samples [33, 34]. The presence of fermentation-associated genera, such as *Ruminococcus* and *Coprococcus* [35, 36], may be consistent with altered community composition, but additional evidence would be needed before interpreting this as dysbiosis. Alternatively, as in the case of *Prevotella*, their presence could result from bacterial migration from the upper to the LRT, or other ecological processes, rather than a defined functional role.

Among genera containing PRDC-associated species, *Mycoplasma*-assigned reads were more prominent at slaughter, whereas *Actinobacillus*-assigned reads were more frequent in nursery and finishing samples, particularly at 100 and 140 days. However, genus-level *16 S-rDNA* data do not allow species-level confirmation or classification of these taxa as members of the normal lower respiratory microbiota in swine. In contrast, sow samples showed a higher number of *Actinobacillus*-assigned reads compared to other genera. It is important to note that the sow farm, nursery, and finishing phases were negative for *Actinobacillus pleuropneumoniae*, although no information was available for other *Actinobacillus* species. On the other hand, *Bordetella* and *Glaesserella* reads were not detected in the analyzed samples. This absence may reflect low abundance, sampling limitations, methodological constraints, or herd-specific circulation patterns; the present study was not designed to determine the cause of their absence.

LEfSe analysis (Fig. 8) indicated that *Bdellovibrionota*, including *Bdellovibrio bacteriovorus*, were enriched in PL− animals. Although *B. bacteriovorus* exhibits predatory activity against some Gram-negative bacteria [36–38], its role in the swine respiratory tract remains unknown; therefore, this finding should be interpreted cautiously. ASVs classified as *Prevotella paludivivens*, also enriched in PL− animals, have been associated with mucosal environments in other body sites [39]; however, their function in the respiratory tract remains unclear. In contrast, ASVs classified as *Tetragenococcus* and *Bacteroides* were associated with the group with lung lesions (PL+) in this data set. However, the present 16 S rRNA gene-based analysis cannot determine whether these taxa contribute to lesion development or reflect secondary changes in the microbial community. Previous studies have described physiological traits of *Tetragenococcus halophilus*, such as tolerance to high NaCl concentrations and production of organic acids [39], and have identified Bacteroides coprosuis as an intestinal-associated species [40]. However, the relevance of these characteristics in the swine respiratory tract remains unclear. Therefore, these findings should be interpreted cautiously and highlight the need for further studies to clarify the functional roles of these microorganisms in the respiratory microbiota.

## Conclusion

This study revealed dynamic changes in the LRT microbiota across swine production phases and between animals with and without lung lesions. We identified candidate discriminant taxa, including ASVs classified as *B. bacteriovorus* and *T. halophilus*, associated with PL- and PL+ groups, respectively. These findings provide insights into longitudinal patterns of the swine respiratory microbiota and highlight potential microbial associations with health and disease. However, given the exploratory nature of this *16 S*-based study, further research is needed to validate these associations and better understand the functional roles of these microorganisms in the respiratory tract. The biological relevance of these associations remains to be clarified.

## Supplementary Information

Below is the link to the electronic supplementary material.

Supplementary Material 1 (XLSX 39.4 KB)
